# What Got You Here, Won’t Help You There: Changing Requirements in the Pre- Versus the Post-tenure Career Stage in Academia

**DOI:** 10.3389/fpsyg.2021.569281

**Published:** 2021-02-04

**Authors:** Stephanie K. Rehbock, Kristin Knipfer, Claudia Peus

**Affiliations:** TUM School of Management, Technical University of Munich, Munich, Germany

**Keywords:** academia, academic career stages, academic gender gap, gender stereotypes, STEM

## Abstract

Despite efforts to foster gender equality in academia, women are vastly under-represented in tenured professorships, specifically in STEM disciplines. While previous research investigated structural and organizational barriers for women in academia, we explored professors’ subjective view on attributes required before and after reaching tenure. The perspective of professors is needed as they are gatekeepers when it comes to the career advancement of junior researchers. Hence, we interviewed 25 tenured STEM professors in Germany about which attributes they personally consider to be required pre- versus post-tenure and analyzed whether these attributes are associated with gender stereotypes. We found that different attributes are mentioned in the pre- versus the post-tenure career stage and that the required attributes can be associated with gender stereotypes: While agentic–stereotypically male–attributes were mentioned more frequently than communal attributes in the pre-tenure career stage, communal–stereotypically female–attributes were reported slightly more often than agentic attributes after reaching tenure. Based on these novel findings, we discuss important implications for gender research and practice to contribute to more diversity and transparency in academic career advancement.

## Introduction

Despite decades of insightful research on academic careers and efforts to counteract gender inequality in academia, women remain under-represented in advanced academic career stages, particularly in the male-dominated STEM (science, technology, engineering, and mathematics) disciplines (Ceci and Williams, 2007; Ysseldyk et al., 2019). Specifically in Germany, one of the most male-dominated academic communities globally (Best et al., 2013), only as little as 13% of STEM professors are women–and this although 35% of Ph.D. candidates in STEM disciplines are female (DESTATIS, 2019). This illustrates that the potential of qualified men and women is not equally exploited throughout their academic career; instead, female talent is left behind (van den Brink and Benschop, 2012, 2014). This is problematic for two reasons: First, more than ever, universities are challenged to attract and retain excellent female scientists in professorships (Blickenstaff, 2005; Moss-Racusin et al., 2012). Second, female scientists face manifold challenges when it comes to advancing to professorship (Zimmerman et al., 2016; Knipfer et al., 2017; Nielsen, 2017). Thus, the main problem we address with this research is the under-representation of women in tenured professorships.

In previous research, the dominant explanation for the under-representation of women in tenured professorships is a perceived *lack of fit* (Heilman, 1983, 2001) for women and professorships, which is based on gender stereotypes, images of the typical leader, and images of the typical scientist (Eagly and Karau, 2002; Deem, 2003; Carli et al., 2016; Herschberg et al., 2018; Teelken et al., 2019). Research on gender stereotypes shows that women are more associated with *communal* attributes (e.g., caring, kind, supportive) than men. In contrast, men are associated more with *agentic* attributes (e.g., competitive, dominant, proactive) than women (Eagly and Karau, 2002; Hentschel et al., 2019). Agentic qualities overlap with stereotypic attributes of academics, who have been described as independent, competitive–and male (Bleijenbergh et al., 2012; Carli et al., 2016). Overall, research on gender stereotypes suggests a better fit for men in professorships and thus higher chances for men to eventually reach tenure (van den Brink and Benschop, 2012). Yet, we know little about what attributes are required in the pre- versus the post-tenure career stage. This research is the first to elucidate the required attributes from the perspective of tenured professors, who are important gatekeepers in academia, and to examine whether these attributes are associated with gender stereotypes.

In Germany, tenured professors solely decide whom to support in their pursuit of an academic career (Harley et al., 2004), and they are decision-makers in appointment procedures for professorships. Hence, their subjectively held expectations should leverage the chances of junior researchers to advance to professorship to a large extent. However, previous studies focused either on formal selection processes and on structural barriers for women to advance to professorships (van den Brink and Benschop, 2012, 2014; Helgesson and Sjögren, 2019) or on explanations why women “opt out” (Nielsen, 2017). Hence, we lack profound insights into what tenured professors expect from junior researchers in terms of required attributes. At the same time, tenured professors can provide realistic insights into required attributes in the tenured stage. Our research seeks to answer the question of *which attributes are required in the pre-tenure versus the post-tenure career stage*. Moreover, we analyze *whether the discovered attributes are associated with gender stereotypes*, which may point to a previously neglected barrier for women in climbing the academic ladder to tenure.

Given that empirical research on required attributes pre-versus post-tenure is scarce, and no previous study has considered the subjective perspective of professors in elucidating these attributes, we used an exploratory approach. Drawing on qualitative semi-structured interviews with tenured professors from various STEM disciplines, we analyzed professors’ subjective view on required attributes pre- versus post-tenure. Based on a qualitative content analysis (Mayring, 2000), we found that professors mentioned different attributes when speaking about the pre-tenure stage than when speaking about the post-tenure stage: Our findings imply that different attributes are required pre- versus post-tenure and that the required attributes are associated with gender stereotypes. Due to the focus on agentic–stereotypically male–attributes in the pre-tenure stage, female researchers may be lost at the early career stage, even though communal–stereotypically female–attributes gain more relevance post-tenure than pre-tenure.

Our study’s contributions are twofold: First, we extend previous research, which has consistently shown that universities are gendered institutions and that gender bias exists on several levels, hindering women from reaching tenure (van den Brink and Benschop, 2012; Treviño et al., 2015; Conesa Carpintero and González Ramos, 2018; Lansu et al., 2019; Teelken et al., 2019). Second, we shed light on the previously neglected senior career stage, which has been described as opaque and predominantly associated with the male stereotype (Bleijenbergh et al., 2012; Evans, 2017; Ooms et al., 2019; Zacher et al., 2019). Our findings offer practical implications for early career support as well as organizational initiatives for the selection, promotion, and development of (female) scholars that aim to contribute to further convergence toward gender balance in academia.

### Think Professor–Think Male? Gender Stereotypes in Academia

The *leaky pipeline* in academia has not been “caulked” in recent years, showing the continued existence of an invisible glass-ceiling, specifically in STEM disciplines (Blickenstaff, 2005; Ysseldyk et al., 2019). Research from the business context has proposed a perceived *lack of fit* between women and senior positions (Heilman, 1983, 2001) as the dominant explanation for the leaky pipeline. Women are generally associated with *communal* characteristics, such as being affectionate, gentle, helpful, and kind, while men are generally said to possess *agentic* qualities, such as being ambitious, assertive, confident, and dominant (Eagly and Karau, 2002). These *agentic* qualities are also attributed to typical leaders, especially in male-dominated fields (e.g., “think engineer–think male phenomenon”; Male et al., 2009). Hence, gender stereotypes–generalizations about women and men based on their gender (Heilman, 2012)–can explain why men are perceived to fit better to leadership positions than women.

Although much research has examined the *lack of fit*-model in the business context, there is empirical evidence pinpointing the same hindrances for women’s career advancement in academia: For example, Fox (2006) found that valued attributes for senior career stages in academia are ascribed more to men than to women. Similarly, the ideal academic is stereotypically described as competitive, lone, and independent (Bleijenbergh et al., 2012). van den Brink and Benschop (2012) highlighted that the commonly held notion of academic excellence reflects the masculine stereotype and Carli et al. (2016) described the stereotypical beliefs in academia as “women ≠ scientists.” The association between men and science is particularly strong in STEM fields (Lane et al., 2012; Eaton et al., 2020). Based on this evidence, we conclude that the stereotype of professors–scientists in senior positions–should also be highly *agentic*, i.e., associated with male qualities: “think professor–think male.”

Overall, the perceived *lack of fit* between women and professorships results in disadvantages that hinders women from applying, being selected, or promoted to professorships (Heilman, 2012; Zimmerman et al., 2016; Zacher et al., 2019). Not only do gender stereotypes result in negative expectations about a woman’s performance in those positions, but concurrently women themselves perceive a lower fit with professorships (Knipfer et al., 2017; Ysseldyk et al., 2019). As a consequence, female researchers often experience a *glass-ceiling* in reaching tenure in academia. This strong influence of fairly stable gender stereotypes (Ibarra et al., 2013; Hentschel et al., 2019) is particularly problematic, because it remains elusive as to whether those stereotypes adequately represent the required attributes in different academic career stages (van den Brink and Benschop, 2012; Evans, 2017). Thus, the key interest of this study is to understand professors’ subjective view on required attributes in two academic career stages, namely pre- and post-tenure, and whether these are associated with gender stereotypes.

### Advancement From the Pre- to the Post-tenure Career Stage

Choosing an academic career is a unique career path with context-specific requirements for each career stage (Zacher et al., 2019). To progress from Ph.D. level to professorship, high discipline-specific expertise and maximization of research output is relevant (Bedeian, 2004; McGrail et al., 2006; Braun et al., 2013; Seibert et al., 2017; Zacher et al., 2019). Only few scholars reach a tenured professorship due to the highly demanding requirements at junior stages. Whilst reaching tenure is a central tenet in an academic career, we know surprisingly little about what attributes are expected of tenured professors (Evans, 2017). The scarce evidence points out that tenured professors not only need to balance research, teaching, and administration, but that they also need to possess leadership skills (Rowley and Sherman, 2003; Bryman, 2007; Braun et al., 2009; Macfarlane, 2011; Evans, 2017; Rehbock, 2020a). Professors supervise junior researchers during their qualification phase, act as mentors, influence others as role models, manage research teams, and develop compelling visions for their group. Concurrently, they are challenged by managing autonomy, change, and uncertainty, even though they have not been systematically prepared for these leadership responsibilities (Smith and Hughey, 2006; Braun et al., 2016; Rehbock et al., 2019). Due to the specifics of the academic system, Lowman (2010) posits that leadership in academia is substantially more complex than leadership in a corporate context. Despite these first insights on the senior career stage, we lack an integrative perspective of different career stages as most research on academic career development has focused on early career stages (Evans, 2017; Ooms et al., 2019; Zacher et al., 2019).

Besides the focus on early career stages, previous investigations have mainly focused on the formal requirements for academic career advancement, such as peer-reviewed publications, networking, and mobility (Baruch and Hall, 2004; Bedeian, 2004; Spurk et al., 2015), and on formal selection processes, such as appointment procedures (van den Brink et al., 2013; Helgesson and Sjögren, 2019). Hence, we lack empirical evidence on the more “informal” expectations for researchers in the pre- versus the post-tenure stage. Specifically, we need to understand what attributes tenured professors consider to be required pre- versus post-tenure, because they act as gatekeepers and decision-makers who select and promote junior researchers.

In summary, this research aims to elucidate (a) the required attributes before versus after reaching a tenured professorship, and (b) whether these attributes are associated with gender stereotypes.

## Materials and Methods

As we are the first to elucidate what attributes are required pre- versus post-tenure from the perspective of professors, we conducted and analyzed 25 semi-structured interviews with tenured STEM professors in Germany.

### Recruitment and Sample Description

We chose German academia as our research context, because post-doctoral researchers can apply directly for full professorships in Germany (Harley et al., 2004). This may lead to greater differences in required attributes pre- versus post-tenure than in other countries, where there are intermediate career stages (e.g., assistant professorships). Furthermore, German academia–and STEM specifically–has been characterized as one of the most male-dominated academic contexts globally (Best et al., 2013). Hence, gender stereotypes should be more pronounced than in less male-dominated contexts (see extreme contexts, Bamberger and Pratt, 2010).

We focused our theoretical sampling strategy (Eisenhardt et al., 2016) on tenured professors for two reasons. First, tenured professors can provide insights into required attributes pre-tenure because their view will determine to a large extent who will advance to more senior stages by mentoring, selecting, and promoting junior researchers’ career progress. Hence, professors’ subjective views on required attributes pre-tenure are highly relevant for the career advancement of junior researchers. Second, tenured professors can provide information as to which attributes are needed to deal with their daily job demands in the post-tenure career stage.

We recruited 25 professors from three research- and technically-oriented public universities with 25,000 to 51,000 students (Times Higher Education, 2019). As the authors are employed at one of the three universities, they are familiar with the specifics of academic careers and the German academic system. The interviewer (first author) had no previous contact to any of the interview partners to ensure impartiality and confidentiality. We aimed at achieving diversity in the study sample with regards to age, gender, fields of research, and years of tenure. We recruited via email, briefly introducing our research interest and requesting a 1-h face-to-face interview. Some interview partners supported us by recruiting additional participants from their own network. Once the statements in the interviews increasingly corresponded with previous interviews, we concluded the data collection (see King and Horrocks, 2010).

The final sample included eight women and 17 men. As women accounted for 32% of our sample, they were over-represented in comparison to the target population. The average age of participants was 54 years, ranging from 39 to 76 years. On average, the participants had completed their doctoral dissertation at the age of 30 (women: 28; men: 31) and had been appointed to tenured professorships 15 years ago (ranging from 2 to 38 years) at the average age of 39 (women: 37; men: 40). Professors came from different STEM fields (see Table 1): Science (eight professors), technology (six professors), engineering (seven professors), and mathematics (four professors).

**TABLE 1:** Overview of sample.

Discipline	Number of interview partners
Science	8 (5 men)
Technology	6 (4 men)
Engineering	7 (6 men)
Mathematics	4 (2 men)

### Data Collection

The first author conducted the semi-structured interviews between December 2016 and January 2018 in German and in person to maintain consistency. The majority of interviews took place in professors’ offices to make our interviewees feel as comfortable and secure as possible to ensure open responses (King and Horrocks, 2010). We aimed at gaining a comprehensive overview of required attributes from both, interviewees’ expectations toward others as well as their personal experience before and after reaching tenure. The interview guideline included three major themes: (1) professors’ work routines, including typical tasks, behaviors and challenges in their day-to-day work; (2) required attributes for junior researchers they supervise (e.g., “When you think about your group, who would you identify as someone who has the potential to become a professor, and why?”) and success factors for their own advancement to professorship (e.g., “What attributes were important for your own career advancement to reach tenure?”); and (3) their subjective perception of required attributes as a professor (e.g., “What does it take to be a professor?”; “What traits and behaviors are important in your current role?”). At the end of each interview, the interviewer requested or verified demographic information, which was researched beforehand on professors’ official websites. The average interview duration was 62 min, ranging from 36 to 118 min. All interviews were audio-recorded and transcribed verbatim (1540 min; 757 pages).

### Data Analysis

We applied qualitative content analysis (Mayring, 2000) to combine the strength of inductive analysis techniques (Glaser and Strauss, 1967; Strauss and Corbin, 1998) to identify emerging themes (=attributes) and traditional content analysis (Krippendorff, 1980) to elucidate attributes required pre- versus post-tenure.

In a first step, the first author analyzed each interview by coding statements about attributes^1^ required in the pre- versus post-tenure career stage using the software program MAXQDA. At this point, 1.440 statements about required attributes were identified across all interviews: 546 statements referring to required attributes in the pre-tenure stage (e.g., “from junior researchers, I expect that they work independently and have an inner drive,” Int. 9) and 894 statements referring to required attributes in the post-tenure stage (e.g., “professors should treat their team fairly and listen to their employees,” Int. 22).

In a second step, we inductively generated higher-order codes (data reduction). To this end, the first author clustered similar statements and assigned higher-order codes that described the content of these statements. For example, we clustered the statement “[when hiring junior researchers], their *achievements* are very important” (Int. 2) and the statement “student’s high *achievements* in their studies are a strong indicator for success” (Int. 28) and assigned the higher-order code (=attribute): *achievement orientation* (pre-tenure). Importantly, in some interviews, multiple statements were made that referred to the same attribute. In the process of clustering and coding statements, the first author discussed the emerging higher-order codes with a second rater and the remaining co-authors in order to establish intersubjective consensus. Overall, 40 higher-order codes (=attributes) were identified at this stage.

In a third step, the discovered attributes were assigned theoretical codes using the widely established dichotomy *agentic* versus *communal* (see Abele and Wojciszke, 2014, p. 196): “Agentic content refers to goal achievement and task functioning (competence, assertiveness, decisiveness), whereas communal content refers to the maintenance of relationships and social functioning (benevolence, trustworthiness, morality).” For example, we coded the attribute *independent* as *agentic*, whereas we coded the attribute *supportive* as *communal* (based on Gaucher et al., 2011; Heilman, 2012; Hentschel et al., 2019; Pietraszkiewicz et al., 2019). A few attributes were coded as *neutral*, such as creative, flexible, or funny. As the number of neutral attributes was negligible and because of our interest in attributes associated with gender stereotypes, we focus on agentic and communal attributes in the description and discussion of our findings. To ensure accurate assignment of theoretical codes, we challenged and verified the assignment of the theoretical codes as either agentic or communal in collaboration with an expert in gender-fair language. The final list of agentic and communal attributes includes 34 attributes (see Appendix A).

The list of 34 agentic and communal attributes was based on the clustering and coding of 1.363 statements: 501 statements in the pre-tenure career stage (402 coded as agentic; 99 as communal) and 862 statements in the post-tenure career stage (356 coded as agentic; 506 as communal). After finalizing the overall frequency analyses per career stage, we complemented our analysis by an additional frequency analysis of required attributes for each career stage per interviewee gender.

In a fourth step, an additional coder, who was blind to our research questions, used the final list of attributes (see Appendix A) to recode 1/3 of the interviews to establish interrater reliability for the assignment of higher-order codes (=attributes). There was an agreement of 91.94% with the first coder, which means near perfect agreement. Finally, we discussed our findings with scholars in the field of gender and academic career research at international conferences, such as the Annual Meeting of the Academy of Management 2019.

## Findings

**FIGURE 1:**
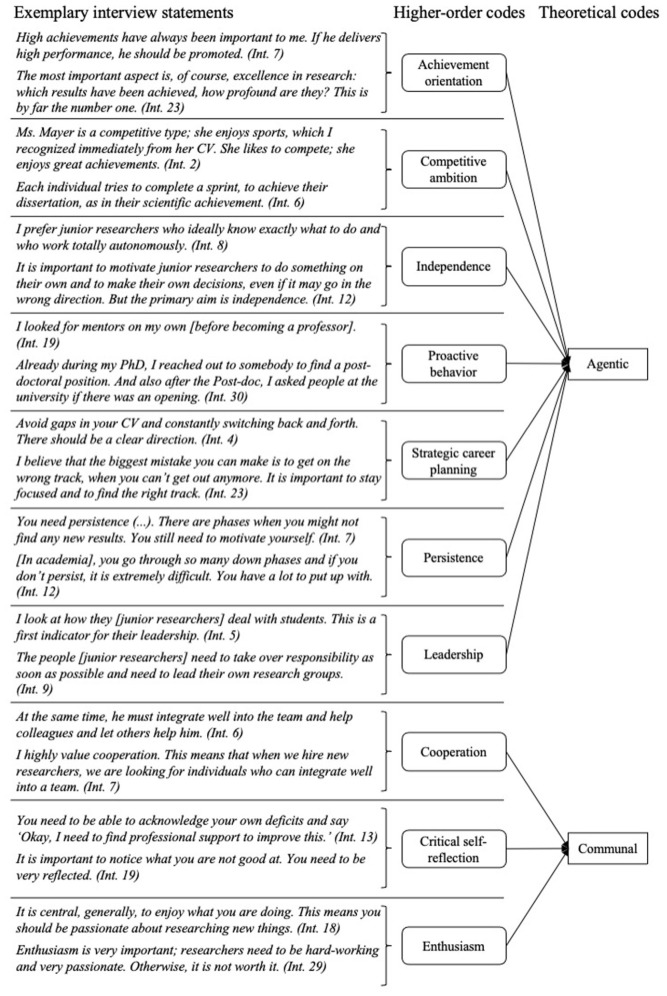
Data structure of the ten most frequently mentioned attributes pre-tenure.

**FIGURE 2:**
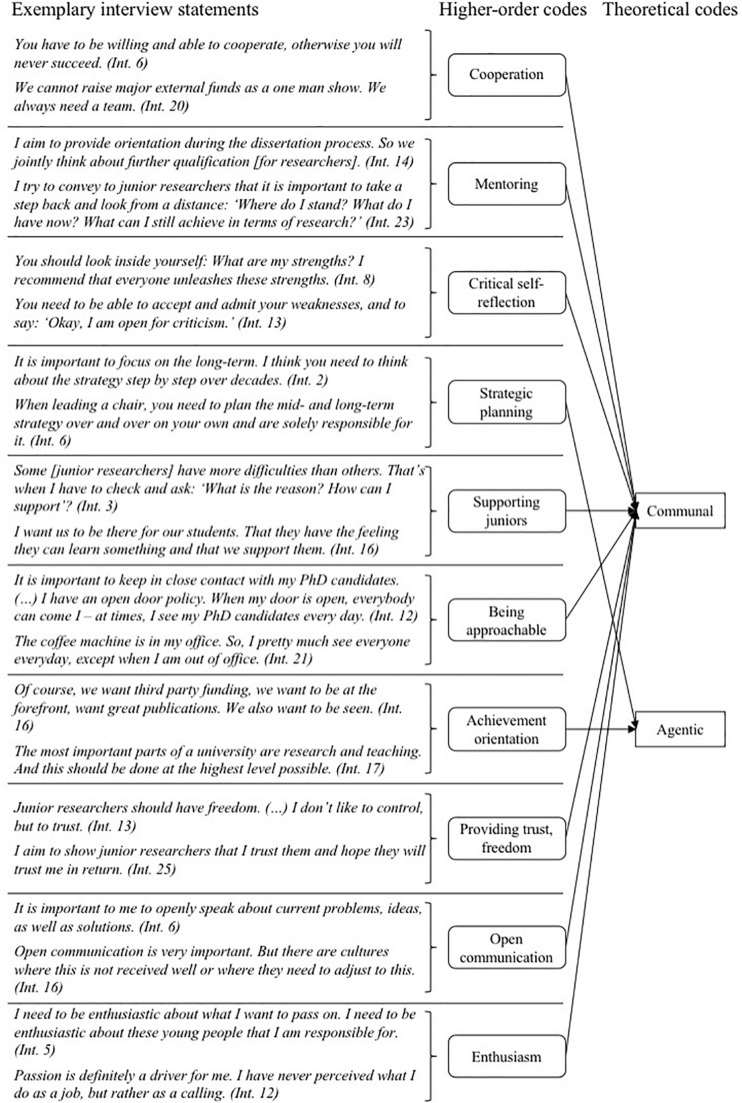
Data structure of the ten most frequently mentioned attributes post-tenure.

The purpose of this study was to elucidate professors’ view on attributes required pre- versus post-tenure and to analyze whether these attributes are associated with gender stereotypes. As a key result, we found that different attributes were mentioned in the pre- versus the post-tenure career stage: Our interviewees mentioned agentic–stereotypically male–attributes more often than communal attributes when asked about the pre-tenure career stage (see Figure 1, e.g., *competitive*, *independent*, *achievement-oriented*). In contrast, when professors spoke about the post-tenure career stage, they mentioned communal–stereotypically female–attributes slightly more frequently than agentic attributes (see Figure 2, e.g., *cooperative*, *supportive*, *helpful*).

The most frequently mentioned attributes will be discussed separately for the pre- versus the post-tenure career stages in the following paragraphs.

### Pre-tenure: Focus on Agentic Attributes

Of all attributes that were required at the pre-tenure stage, 80.2% were coded as agentic, such as *competitive*, *proactive*, or *strategic planning* (see Table 2 for the ten most frequently mentioned attributes).

**TABLE 2:** Overview of ten most frequently mentioned attributes pre-tenure.

#	Required attributes pre-tenure	Dimension	Absolute frequency	Relative frequency of pre-tenure attributes
1	Achievement orientation	Agentic	79	15.8%
2	Competitive ambition	Agentic	62	12.4%
3	Independence, self-management	Agentic	61	12.2%
4	Proactive behavior	Agentic	44	8.8%
5	Strategic (career) planning	Agentic	33	6.6%
6	Persistence	Agentic	31	6.2%
7	Leadership	Agentic	27	5.4%
8	Cooperation	Communal	24	4.8%
9	Critical self-reflection	Communal	22	4.4%
10	Enthusiasm	Communal	21	4.2%

In the following paragraphs, we will exemplify the three most frequently mentioned attributes that professors considered to be required at the pre-tenure stage: *achievement orientation* (79; 15.8%)^2^, *competitive ambition* (62; 12.4%), and *independence* (61; 12.2%).

#### Achievement Orientation

The majority of professors emphasized the importance of achievement orientation for an academic career, as Prof. 23 explained: “The most important aspect is, of course, excellence in research: which results have been achieved, how profound are they? This is by far the number one.” In order to fulfill these high requirements, an engineering professor (Prof. 3) further explained:

We search for individuals who are not only interested in finding the fastest solution, but who are motivated to orientate themselves on basic research and substantially contribute to a problem’s solution. (Prof. 3).

An informatics professor (Prof. 18) added that he had extremely high expectations but that, in return, he offered excellent education for students’ future careers. Another informatics professor (Prof. 1) reported that, in her career, excellent performance had always compensated for her low self-confidence: “[After finishing my studies,] I was offered a research associate position. Apparently, I caught enough attention based on my performance, even though I never actively pushed for it or screamed “here.” She elaborated that she had always worked extremely hard: “During my mathematics studies, I repeated the past semester’s content in every break, worked through all books, and looked into forthcoming course content.”

When deciding on whom to supervise for a doctoral thesis, several professors stated that they mainly looked at previous achievements, such as the grade point average of their studies or the evaluation of their master thesis. Prof. 9 explained: “The best indicator or predictor for future performance is past performance. That’s why I aim to understand an applicant’s past performance.”

#### Competitive Ambition

During the interviews, professors explained that they considered it an important attribute for junior researchers to be competitive–and even selfish–to succeed in academia, as Prof. 2 emphasized: “Ms. Mayer is a competitive type; she enjoys sports, which I recognized immediately from her CV. She likes to compete; she enjoys great achievements.” A physics professor (Prof. 11) further emphasized the importance of extremely strong ambition: “I want [junior researchers] to show the willingness to work so hard, that they try to become the best worldwide in a specific domain–the first and the best.” Prof. 20 stated that an academic career was a never-ending competition. A physics professor (Prof. 17) explicated that speed and ambition was important, considering the risk that another research group may be pursuing the same research and succeed faster:

If anyone does the same research faster, it was all for nothing. It’s a tough competition, and I tell students who come to me that they have to face this competition. The best [students] are then challenged by this, and I enjoy collaborating with these kinds of people. (Prof. 17).

The important role of competition was further highlighted by an informatics professor (Prof. 24) who stated that the evaluation of research output had increasingly been conducted by excluding the negative: “The lower the acceptance quote, the better the conference.”

A chemistry professor (Prof. 9) further explained that competitive ambition was a crucial success factor for an academic career: “Something that all great people have in common is an inner drive to accomplish something great, and the ambition to achieve a successful project.” Ambition and grit were emphasized in several interviews, for instance, by a mathematics professor (Prof. 19), who explained that scholars who left academia might not possess the grit and ambition needed to take sacrifices: “You need grit, this very great ambition, and, obviously, there is a pay-off: You can make more money elsewhere and have more security, but this is not important to me.” Similarly, an engineering professor (Prof. 12) stated that, for him, it was most important to see the “burning fire of ambition for their work.” Prof. 16 explained that, in his view, this strong focus on ambition is an essential part of pursuing a Ph.D. He believed that this competitive ambition “pushes junior researchers in the direction of doing everything on their own.”

#### Independence and Self-Management

The third most frequently mentioned attribute for junior researchers was the ability to work independently and to manage one’s time and resources effectively. Prof. 3 stated:

It is important that junior researchers recognize what needs to be done, and that they self-initiate such. This means assuming responsibility on their own and not only waiting to be put in charge. (Prof. 3).

Several professors stated that they preferred working with junior researchers who act completely autonomously and do not need detailed guidance, for instance, Prof. 8: “To be honest, I prefer researchers who ideally know exactly what to do and who work totally autonomously, so that all I have to do is confirm: *Great, that’s done*.” As far as unexperienced Ph.D. candidates are concerned, professors saw it as their task to foster their independence and self-management skills. For example, Prof. 3 elaborated: “In the long run, my idea is to “force” or educate junior researchers to work autonomously.”

The reasons for emphasizing independence varied across interviews. Some professors considered it to be the general key to success for an academic career. Specifically, post-doctoral researchers needed to show the ability to work autonomously and to take responsibility for their own work to be prepared for their academic career. Another professor (Prof. 9) highlighted:

People who cannot imagine permanently working for somebody else–that’s the best predictor for an academic career, complemented by an inner drive. People who say: “I fit in well and I am team-oriented, it’s all the same to me if I receive instructions, I even like it because I don’t have to take over responsibility”–these people feel more comfortable in industry. Professors are individualists. (Prof. 9).

Other professors expected high autonomy from junior researchers because it facilitated and relieved their own effort in supervising them; as Prof. 24 put it: “I don’t like Ph.D. students who are extremely good but need a lot of guidance or don’t dare to follow their own ideas. They are the least pleasant.” Professors preferred to recruit Ph.D. candidates from their undergraduate students or research assistants who had already demonstrated their ability to work autonomously. An engineering professor (Prof. 3) concluded: “You need to prove that you managed yourself in overcoming difficulties, in searching for alternative solutions, ways to solve problems that were not given.”

In summary, the three most frequently mentioned attributes for junior researchers (*achievement orientation*, *competitive ambition*, *independence*) can be categorized as agentic–stereotypically male–attributes. When comparing the responses of male and female interviewees, the proportion of attributes classified as communal or agentic was similar to the overall proportion: Of the pre-tenure attributes that women provided, 80.7% were coded as agentic attributes. Men’s statements on pre-tenure attributes that were coded as agentic accounted for 79.9%. Yet, women and men differed slightly in how often specific attributes were mentioned (see Table 3): For example, men mentioned *independence* (45; 14.8%) more often than *competitive ambition* (35; 11.5%), whereas women mentioned *competitive ambition* (27; 13.7%) more often than *independence* (16; 8.1%). Furthermore, female professors mentioned *critical self-reflection* (14; 7.1%) and *enthusiasm* (11; 5.6%) more often than *cooperation* (4; 2.0%), whereas male professors mentioned *cooperation* (20; 6.6%) more often than *enthusiasm* (10; 3.3%) and *critical self-reflection* (8; 2.6%). Because women and men largely mentioned similar proportions of agentic and communal attributes, the overall association between required attributes in the pre-tenure career stage and gender stereotypes seems not to be related to interviewee gender.

**TABLE 3:** Overview of ten most frequently mentioned attributes pre-tenure per gender.

#	Women frequency		Men frequency		Total frequency	
1	Achievement orientation	29	Achievement orientation	50	Achievement orientation	79
2	Competitive ambition	27	Independence, self-management	45	Competitive ambition	62
3	Proactive behavior	18	Competitive ambition	35	Independence, self-management	61
4	Independence, self-management	16	Proactive behavior	26	Proactive behavior	44
5	Critical self-reflection	14	Strategic (career) planning	20	Strategic (career) planning	33
6	Strategic (career) planning	13	Persistence	20	Persistence	31
7	Persistence	11	Cooperation	20	Leadership	27
8	Enthusiasm	11	Leadership	17	Cooperation	24
9	Leadership	10	Enthusiasm	10	Critical self-reflection	22
10	Emotional Strength	10	Emotional Strength	9	Enthusiasm	21

*Interviews Women: n = 7. Interviews Men: n = 18.*

### Post-tenure: Focus on Communal Attributes

We found that the required attributes change toward more communal attributes when it comes to the post-tenure stage. Of all attributes that were considered to be required at the post-tenure stage, 58.7% were coded as communal, e.g., *cooperative*, *caring*, and *supportive* (see Table 4 for the ten most frequently mentioned attributes).

**TABLE 4:** Overview of ten most frequently mentioned attributes post-tenure.

#	Required attributes post-tenure	Dimension	Absolute frequency	Relative frequency of post-tenure attributes
1	Cooperation	Communal	62	7.2%
2	Mentoring	Communal	54	6.3%
3	Critical self-reflection	Communal	54	6.3%
4	Strategic planning	Agentic	53	6.1%
5	Supporting and helping junior researchers	Communal	53	6.1%
6	Taking time for team, being approachable	Communal	46	5.3%
7	Achievement orientation	Agentic	45	5.2%
8	Trusting and giving freedom to others	Communal	39	4.5%
9	Open communication	Communal	35	4.1%
10	Enthusiasm	Communal	32	3.7%

In the following paragraphs, we explain the three attributes most frequently mentioned by professors for the post-tenure career stage: *cooperation* (62; 7.2%)^3^, *mentoring* (54; 6.3%), and *critical self-reflection* (54; 6.3%).

#### Cooperation

Once a tenured professorship is reached, professors highlighted the importance of being a good team-player. Prof. 6 emphasized that cooperativeness is the key for every research project:

You have to be willing and able to cooperate, otherwise you will never succeed in a joint research proposal, and you will never be fully accepted in the scientific community. The scientific community is a mix of strong competition and great respect for other researchers. (Prof. 6).

Several interviewees stated that cooperation was key in achieving research success and that they put high priority on a collaborative climate by introducing flat hierarchies and team-building activities for their research groups. Prof. 14 explained “Everybody should have the feeling of being part of this team. This is extremely important.” Moreover, Prof. 10 stated: “We are all leading [scientists], because we have the right people.” The importance of collaboration for knowledge-sharing was also mentioned, as expertise was usually distributed among researchers in a group. Prof. 5 stated that he sought advice from his research group when making decisions about the research strategy:

I am way too insecure about my own position to solely determine where to go (…) I would never force my team to be interested in a topic, just because it was useful for me. (Prof. 5).

An informatics professor (Prof. 18) added that research was becoming increasingly interdisciplinary: “In fact, we have to collaborate with everybody to achieve progress in important fields.” Due to the importance of collaboration for research progress, Prof. 8 stated that being able to cooperate was a key criterion in appointment procedures for full professors: “People are selected based on their willingness to cooperate. The university wants to know: is this woman or man open to collaboration? If not, they are not chosen.”

#### Mentoring

Next to cooperation, the second most frequently mentioned attribute in the post-tenure stage was mentoring, especially when leading and supervising junior researchers and accompanying their scientific progress. A mathematics professor (Prof. 7) stated: “I try to nurture my students’ competencies, so that they develop further.” Several professors stated that they held individual (bi-)annual feedback sessions with every member of their research group and the junior researchers they supervised. They would reflect on the past year, discuss personal career goals and how to achieve those goals. A mathematics professor in her forties explained (Prof. 19): “My generation of professors asks junior researchers: “What do you need?,” or we offer: Would you prefer x or y, and then they can choose.” Similarly, Prof. 7 stated:

I try to help my students to forge their own path, wherever it is. As a mentor, I don’t only focus on getting the best [Ph.D.] students, but I consider carefully what is best for them. I want to coach them toward having all the opportunities they want and not having to decide whether they want to have a family and should therefore not pursue their Ph.D., or similar choices. (Prof. 7).

Besides individual mentoring of junior researchers, professors highlighted their role as mediators in the case of conflicts. An informatics professor (Prof. 16) explained: “Whenever there is an issue, I try not to take sides, but listen instead.” He further explained that he usually tried to mediate as a mentor and “not to act like the big boss.” His objective was to discuss among equals and to share best practices which had supported his own career. An engineering professor (Prof. 20) explained that it was of utmost importance for junior colleagues to learn from his experience, and, for him, to pass on his experience to them, not only knowledge-wise, but also in respect of personal development.

#### Critical Self-Reflection

As the third most frequently mentioned attribute in the post-tenure stage, interview partners emphasized the importance of taking time to self-reflect and to seek feedback. Prof. 8 explained: “You have to look inside yourself: What are my strengths? I recommend that everyone unleashes these strengths.” Another professor (Prof. 4) explained that she gained thorough feedback from a life coach she met bi-weekly. Besides knowing one’s strengths and weaknesses, interviews illustrated that it was also important to reflect on one’s position within the university. For instance, Prof. 12 explained:

In the long run, we have to ask ourselves: “Do we agree with the current university and our system?” I think, specifically in academia, it is important that we question ourselves and the structures which have developed over decades. To me this is part of academic self-conception, that you take nothing as a given. (Prof. 12).

An informatics professor (Prof. 1) added that it was important to ask yourself how to deal with your profession, as in questioning “Is this how I want to be? Do I want to work in the lab or on the computer every day from 8 am until midnight? Or do I want to pursue other priorities in my life, whether this is a relationship, family, friends, or a hobby?” She found it difficult to find answers to these questions, specifically whether there is a need to be constantly available. For example, she struggled with balancing the focus on excellence in her profession and finding time for other important aspects of her life. Reflecting on one’s higher-level goals was further considered to be important to not regret decisions later on. Relatedly, an engineering professor (Prof. 22) explained:

It is important to be able to let go and be open, even though it might be painful. It is not easy to admit that you were wrong, to say “I can’t afford this anymore,” or “We need to change our direction.” To be able to take a step back and follow a new route is not easy. I wish we had more time to think about the next steps. Often, we are pushed into *ad hoc* decisions, because time is ticking. (Prof. 22).

Several professors emphasized the crucial role of feedback for self-development. For instance, an engineering professor (Prof. 13) decided to hire a coach to video-record his lecture and give him feedback, because he had never received formal training. Another professor (Prof. 1) explained that she used to receive low teaching evaluations and now tries to have closer interaction with students in small learning groups in an effort to understand their challenges and ways of thinking to improve her teaching. At the same time, several interview partners believed that some professors were reluctant to participate in coaching programs or formal training, because “they are afraid to make a fool of themselves or show weaknesses” (Prof. 13).

In summary, the three most frequently mentioned attributes for tenured professors (*cooperation*, *mentoring*, *critical self-reflection*) can be categorized as communal–stereotypically female–attributes. As for the pre-tenure career stage, there were only few differences between the responses of women and men in our interviews. Of all statements that women provided for the post-tenure career stage, 60.8% were coded as communal. Men’s statements that were coded as communal accounted for 57.7%. Similar to the pre-tenure findings, women and men differed slightly in the frequency of required attributes they mentioned (see Table 5): For example, female professors mentioned *supporting and helping junior faculty* (22; 7.9%) most often, whereas *cooperation* (15; 5.4%) was only named as the fifth most frequently attribute. In contrast, male professors mentioned *cooperation* most frequently (47; 8.0%). Overall, the association between required attributes post-tenure and gender stereotypes seems not to differ per interviewee gender.

**TABLE 5:** Overview of ten most frequently mentioned attributes post-tenure per gender.

#	Women frequency		Men frequency		Total frequency	
1	Supporting, helping junior researchers	22	Cooperation	47	Cooperation	62
2	Strategic planning	19	Mentoring	36	Mentoring	54
3	Mentoring	18	Critical self-reflection	36	Critical self-reflection	54
4	Critical self-reflection	18	Strategic planning	34	Strategic planning	53
5	Cooperation	15	Taking time for team, being approachable	33	Supporting, helping junior researchers	53
6	Achievement orientation	15	Supporting, helping junior researchers	31	Taking time for team, being approachable	46
7	Open communication	15	Achievement orientation	30	Achievement orientation	45
8	Trusting and providing freedom to others	14	Trusting and providing freedom to others	25	Trusting and providing freedom to others	39
9	Taking time for team, being approachable	13	Persistence	24	Open communication	35
10	Patience	13	Enthusiasm	22	Enthusiasm	32

*Interviews Women: n = 7. Interviews Men: n = 18.*

To conclude the presentation of our major findings, our interviewees mentioned various required attributes for the pre- and the post-tenure career stage and changing frequencies thereof (see Table 6). By analyzing the attributes according to gendered wording, this study further revealed that the required attributes can be associated with gender stereotypes.

**TABLE 6:** Change of total frequencies of required attributes pre-tenure versus post-tenure.

#	Required attribute	Dimension	Pre-tenure		Post-tenure	Total
1	Achievement orientation	Agentic	79	↘	45	124
2	Independence, self-management	Agentic	61	↘	30	91
3	Competitive ambition	Agentic	62	↘	27	89
4	Cooperation	Communal	24	↗	62	86
5	Strategic (career) planning	Agentic	33	↗	53	86
6	Critical self-reflection	Communal	22	↗	54	76
7	Proactive behavior	Agentic	44	↘	31	75
8	Persistence	Agentic	31	↘	30	61
9	Mentoring	Communal	–	↗	54	54
10	Enthusiasm	Communal	21	↗	32	53
11	Supporting, helping junior researchers	Communal	–	↗	53	53
12	Leadership	Agentic	27	↘	24	51
13	Open communication	Communal	14	↗	35	49
14	Taking time for team, being approachable	Communal	–	↗	46	46
15	Trusting and giving freedom to others	Communal	–	↗	39	39

## Discussion

Despite the numerous efforts to counteract gender inequality, universities lose female talents on their way upward and, as a consequence, women remain under-represented in tenured professorships (Blickenstaff, 2005; Moss-Racusin et al., 2012). This study constitutes one of the first to examine the informally required attributes for an academic career from tenured professors’ subjective view, whilst previous research has focused on structural barriers for the advancement of women to professorships, for instance, by investigating formal criteria in recruitment processes and systematic biases in performance evaluations (e.g., van den Brink and Benschop, 2012, 2014; Spurk et al., 2015; Helgesson and Sjögren, 2019; Teelken et al., 2019; Wiedman, 2019; Eaton et al., 2020).

We sought to answer the questions (a) what attributes are required in the pre- versus the post-tenure career stage and (b) whether these attributes are associated with gender stereotypes. To this end, we conducted 25 semi-structured interviews with tenured STEM professors in Germany to elucidate which attributes they consider to be required before and after reaching tenure. As a main finding, professors mentioned agentic attributes more frequently than communal attributes in the pre-tenure stage and communal attributes slightly more frequently than agentic attributes in the post-tenure stage. Our findings suggest that there is a change in expectations, from a pre-tenure *ambitious* “*agentic*” *junior researcher* to a post-tenure *supportive* “*communal*” *professor*. More specifically, communal attributes were hardly mentioned to be required pre-tenure, but were mentioned with a considerably higher frequency post-tenure than pre-tenure. Importantly, professors still mentioned agentic attributes to be required post-tenure, but to a lesser extent than pre-tenure and less frequently than communal attributes. Hence, we cannot conclude from our data that agentic attributes are less important post-tenure than pre-tenure. Our findings have important implications for theory and practice.

### Contributions

We contribute to the literature in two ways: First, this study advances our understanding of why women remain under-represented in tenured professorships. Adding to previous literature that has linked the stereotype of scientists to the stereotype of men (see Bleijenbergh et al., 2012; Carli et al., 2016; Eaton et al., 2020), we found that the required attributes in the pre-tenure phase are associated with the male stereotype, providing new evidence for the *lack of fit*-model at early career stages in academia (Heilman, 2001). Hence, our findings imply that structural barriers for female researchers are reproduced in professors’ subjective views of what attributes are required for an academic career. Despite striving for objectivity in selection and promotion processes, professors seem to be influenced by the strong association between stereotypically male attributes and academic success at the junior career stage. This also implies that a comprehensive explanation for the gender gap in academia should consider more explicitly the informal role of tenured professors as gatekeepers for the advancement of (female) junior researchers. Our findings may also complement research on gender differences in academic networks and their influence on subjective career success. For instance, Spurk et al. (2015) showed differences in women’s and men’s professional networks and point to the fact that men’s networks consist of a higher proportion of male to female supporters. This, in turn, was positively related to subjective career success. While Spurk et al. (2015), focused on characteristics of the network as contextual factors, we focused on individual attributes (traits and behaviors). Both research streams contribute to a more complete understanding of the complex dynamics leading to the underrepresentation of women in tenured professorships.

Second, going beyond previous research, our study implies that communal attributes (e.g., cooperation with multiple stakeholders, mentoring junior researchers, and critical self-reflection) gain more relevance post-tenure than pre-tenure. This finding contradicts the predominant stereotype of professors, who have been portrayed as dominantly agentic, as yet (“think professor–think male”; see Bleijenbergh et al., 2012; Carli et al., 2016), and suggests a good *fit* between communal–stereotypically female–attributes and the required attributes for tenured professors. The change in required attributes, which we observed in our study, may be related to the different role expectations in early versus later career stages in academia: For example, professors’ role post-tenure may change toward mentoring and caring for others, which may explain why our interviewees considered many communal attributes as required for their own career stage (see also Rehbock et al., 2019). A further explanation may be that professors mentioned more communal than agentic attributes in the post-tenure stage because they assume that researchers have already proven their agentic attributes to reach tenure. By highlighting that communal attributes are equally–or even more–required at the tenured career stage, we are adding to research on different academic career stages, especially at the previously neglected senior career stage (Ooms et al., 2019; Zacher et al., 2019). We thereby shed light on the opacity of the professorial role (Evans, 2017) and provide more transparency on the required attributes as a tenured professor.

### Challenges for Professors Due to the Discrepancy Between Required Attributes

The observed change in frequency of required attributes in the pre- versus post-tenure stage was related to several challenges for professors as they explained in the interviews: Professors had been fostered to show agentic–stereotypically male–attributes to reach success at earlier stages in their careers. However, a lack of preparation in terms of communal capabilities became evident once they had reached tenure. Specifically, our interview partners mentioned that they often faced role conflicts, for instance when feeling the need to gain a competitive advantage over other research groups but at the same time being asked to cooperate with others. Similarly, professors seemed to be insecure about how to lead and supervise their research groups effectively (e.g., authoritative versus participative), even though they emphasized communal attributes for the senior career stage. Related to this finding, previous research from the business context increasingly postulates a *female leadership advantage* in senior career stages because women are more likely to lead in ways that have been shown to be effective (e.g., transformational leadership; Eagly and Carli, 2003; Eagly, 2007). It should be noted that not only women benefit from a communal leadership advantage but that male leaders may benefit even more from displaying communal behaviors (*communality bonus effect*; Hentschel et al., 2018). The insecurity of professors regarding their leadership role highlights both the relevance of leadership in the academic context and the lack of preparation for leadership responsibilities (see also Bryman, 2007; Morris, 2012; Braun et al., 2016; Evans, 2017; Rehbock, 2020a).

### Limitations and Future Research

Our study provides interesting findings and relevant implications that add to a more complete understanding of why women are still underrepresented in professorships. Yet, we are also aware of the limitations of our qualitative approach. Most importantly, generalizability of our findings is limited due to the sample size which is smaller than in quantitative studies. At this stage, a qualitative approach was appropriate to gather rich insights into required attributes in different career stages: Throughout the interviews, we discussed with our interviewees their experience and expectations, giving them the opportunity to explicate and elaborate on the attributes that they consider to be required pre- versus post-tenure. Moreover, we used the opportunity to ask follow-up questions and to compare and contrast different attributes in the interviews. Still, to test the generalizability of our findings, future research is required that uses quantitative data. A reviewer pointed to the possibility to apply automatized text analysis to examine data from much larger samples, and we encourage researchers to consider this option as an avenue for future research.

Additionally, generalizability of our findings may be limited to the specific context of our study, namely STEM-disciplines in German research-oriented universities. We chose this context because of the great difference between the academic career stages pre- and post-tenure and because of the severe under-representation of female researchers in STEM professorships in Germany. These two factors may have contributed to particular salient differences between career stages and differences related to gender stereotypes. Future research should investigate whether our findings are generalizable to other contexts (e.g., academia in Netherlands, United States), where there are various intermediate career stages such as assistant and associate professorships. We further chose STEM-disciplines because the under-representation of female researchers is most salient in these fields and gender stereotypes may be particularly present. While we acknowledge that female STEM professors were over-represented in our sample, we only found negligible differences between female and male interviewees’ responses. Future research should also explore whether the association between required attributes and gender stereotypes exists in other academic disciplines (e.g., humanities or social sciences).

Although we gained new insights on professors’ subjective perspective on which attributes are required throughout an academic career, it might be fruitful to investigate junior researchers’ perspectives on what they consider as required in their own role as well as attributes they expect from tenured professors. Comparing and contrasting different perspectives could be a valuable next step to expand our findings. Another interesting sample could be researchers who left the academic system. Their understanding of required attributes for an academic career may complement the perspective taken in this study.

### Practical Implications

Our findings have several practical implications for the selection, promotion, and development of (female) academics, particularly in the STEM fields, where there is a general shortage of female talent (Xue and Larson, 2015) despite their importance for innovation and global competitiveness (Beede et al., 2011). First, it is of particular importance to make decision-makers aware of stereotypes as well as the particular gender biases that discriminate (female) researchers in selection and promotion processes. Universities should have measures in place to train decision-makers regarding the relevant attributes for different academic career stages. Most importantly, raising awareness about the change of required attributes from agentic to communal throughout an academic career might result in more valid–that is, less biased–evaluations of (female) researchers in tenure decisions. Second, our findings point to the need to promote researchers with both agentic *and* communal attributes–independent of their gender–into professorships. Our findings imply that individuals, who are promoted to tenured professorships based on agentic attributes, may lack the attributes that are necessary after tenure. Hence, criteria for academic promotion should be expanded to prevent an overemphasis of agentic attributes. Specifically, we suggest a stronger consideration of communal attributes when selecting candidates for professorships (at all levels). Third, personnel development should foster both agentic and communal attributes. Leadership development programs for researchers should focus on communal qualities early on to effectively prepare them for the demanding role as professors. Similarly, tenured professors should be supported in balancing and integrating seemingly conflicting–agentic versus communal–attributes.

## Conclusion

This research contributes to the call for more nuanced explanations for the low proportion of female researchers who eventually reach tenure as a professor. Interviews with tenured professors showed that different attributes seem to be required pre- versus post-tenure and that these attributes can be associated with gender stereotypes: More specifically, agentic (stereotypically male) attributes were more frequently mentioned than communal attributes in the pre-tenure stage, while communal (stereotypically female) attributes were mentioned slightly more frequently than agentic attributes in the post-tenure stage. Our findings expand research on why women remain under-represented in professorships and demonstrate that communal attributes gain more relevance in the post-tenure versus the pre-tenure career stage. Our findings suggest that female researchers might be lost due to a focus on agentic attributes before tenure–and this although communal attributes seem to be required in tenured professorships. If agentic stereotypes continue to influence how professors are selected and evaluated, the academic glass-ceiling for women researchers will not break in the near future. We hope that this research stimulates critical reflection on the success factors that shape an academic career and the implementation of gender-fair criteria for tenure decisions.

## Data Availability Statement

The datasets generated for this study are available on request to the corresponding author.

## Ethics Statement

Ethical review and approval was not required for the study on human participants in accordance with the local legislation and institutional requirements. Written informed consent for participation was not required for this study in accordance with the national legislation and the institutional requirements. Oral informed consent for participation and approval by the participants was carried out before the interview and is recorded.

## Author Contributions

SR made substantial contributions to the conception and design of the work, plus the acquisition, analysis, and interpretation of data for the work, drafted the work for critically important intellectual content, and served as both corresponding and first author on the submission. KK made substantial contributions to the conception and design of the work, plus the analysis and interpretation of data for the work, revised the manuscript, and served as second author on the submission. CP made substantial contributions to the conception and design of the work, plus the interpretation of data for the work, provided the editorial guidance, and served as third author on the submission. All authors contributed to the article and approved the submitted version.

## Conflict of Interest

The authors declare that the research was conducted in the absence of any commercial or financial relationships that could be construed as a potential conflict of interest.

## Footnotes

1 Attributes comprised both personal traits and behaviors. We did not code contextual factors (e.g., prestige of institution).

2 *Attribute* (absolute number of statements; percentage of all pre-tenure statements).

3 *Attribute* (absolute number of statements; percentage of all post-tenure statements).

## Appendix A

**TABLE A1:** Coding scheme*.

Higher-order code	Agentic	Communal
Achievement orientation	x	
Appreciative		x
Authentic		x
Authoritarian	x	
Committed		x
Competitive ambition	x	
Confidence	x	
Cooperation		x
Courage, risk-taking	x	
Critical self-reflection		x
Diplomatic		x
Direct communication	x	
Egoistic	x	
Emotional strength	x	
Empathy		x
Enthusiasm		x
Honesty		x
Independence, self-management	x	
Leadership	x	
Mentoring		x
Open communication		x
Participative		x
Patience		x
Persistence	x	
Proactive behavior	x	
Reliablity	x	
Responsibility	x	
Social competence		x
Strategic (career) planning	x	
Supporting and helping junior faculty		x
Strict	x	
Taking time for team, being approachable		x
Trusting and providing freedom to others		x
Value orientation		x

**This list only comprises agentic and communal attributes, as referred to in the section “Data Analysis.”*
